# Effects of irrigation scheduling methods and blended NPS fertilizer on tuber yield and water productivity of potato (*Solanum tuberosum* L.) in northwest Ethiopia

**DOI:** 10.1016/j.heliyon.2023.e19762

**Published:** 2023-09-01

**Authors:** Melkamu Alemayehu, Minwyelet Jemberie, Yigizaw Dessalegn

**Affiliations:** a College of Agriculture and Environmental Sciences, Bahir Dar University, Bahir Dar, Ethiopia; b Woramit Vegetable and Fruit Research Sub-center, Amhara Region Agricultural Research Institute, Ethiopia; c Interational Livestock Research Institute - Livestock and irrigation value chains for Ethiopian smallholders (LIVES) Project, Bahir Dar, Ethiopia

**Keywords:** Partial factor productivity, Plant height, Tuber yield, Tuber weight, Water productivity

## Abstract

Irrigation water scheduling methods influence the growth, yield, and water productivity of crops including potatoes. Therefore, a study was conducted to evaluate the effects of irrigation frequency determination methods on tuber yield, irrigation water productivity and fertilizer use efficiency of potatoes at Koga irrigation scheme, in Northwest Ethiopia. The treatments were consisted of two irrigation frequency determination methods (wetting front detector and crop water requirement) and six NPS fertilizer rates (0, 90.8, 136.2, 181.6, 227.4 and 272.0 kg ha^−1^, which were factorial combined in a randomized complete block design with three replications. Growth and tuber yield data of potato were collected based on the standard procedure. Irrigation water productivity, partial factor productivity, and agronomic efficiency were calculated using their respective models. The collected data were analyzed using SAS version 9.4. The results revealed that the wetting front detector method recorded the highest tuber weight (79.5 g), tuber yield (41.9 t ha^−1^), and irrigation water productivity (9.1 kg potato m^−3^ water) compared to crop water requirement method. NPS fertilizer at the rate of 272 kg ha^−1^ also produced the highest tuber weight (86.5), tuber yield (58.1 t ha^−1^) and irrigation water productivity (12.4 kg potato m^−3^ water). Treatment combination of wetting front detector and 272 kg ha^−1^ NPS recorded the highest plant height (64.m cm) and stem number (10.4). Wetting front detector method recorded the highest partial factor productivity (275.2 kg potato per kg NPS) compared to crop water requirement. Wetting front detector combined with NPS rates generally recorded higher partial factor productivity compared to the respective NPS rates combined with crop water requirement. NPS fertilizer at 272 kg ha^−1^ combined with wetting front detector gave the highest net benefit (236,591.7 ETB ha^−1^) with acceptable marginal rate of return (248.9%), which is recommended for economical production of potato in the area.

## 1 Introduction

Crop production in Ethiopia is dominated by small-scale farming system, which is highly susceptible to rainfall variability both in magnitude as well as occurrence [1]. On the other hand, increasing production and productivity of crops to feed an ever increasing population is the prime concern of developing countries that necessitates the rapid expansion of irrigated agriculture. Accordingly, various large, medium, and small sized irrigation schemes have been developed in the last years in Ethiopia [2].

In irrigation schemes of Ethiopia, large amount of water is wasted due to inefficient delivery and on-farm water management practices [3]. Agide et al. [4] in this regard reported the loss of irrigation water in Meki and Koga Irrigation Schemes in relation to conveyance and distribution systems. The schemes are the major vegetable producing areas of the Ethiopia. Such poor water management practices are partially contributing to the low yield and water productivity observed in irrigation schemes where furrow irrigation is predominantly practiced. On the other hand, climate change manifested in the form of rainfall variability, drought, and high temperature is today more evident than before throughout the world including in Ethiopia [5]. This and the above-mentioned scenarios call for efficient utilization of natural resources including irrigation water. Therefore, the implementation of appropriate on-farm irrigation management practices including irrigation scheduling is quite necessary to meet the ever increasing food demand observed in the country.

Irrigation scheduling is made often using soil water measurement, soil water balance calculations, and plant ‘stress’ sensing [6,7]. Most of the techniques are however relatively difficult to implement practically in the production field at the smallholder level. Stirzaker et al. [7] reported that only less than 15% of farmers in Australia used science-based tools, whereas over 90% relied heavily on “local knowledge”. Taking this into consideration, a device known as Wetting Front Detector (WFD) has been developed and patented by CSIRO Land and Water in Australia.

The WFD comprises a specially shaped funnel, a filter, and a mechanical float mechanism. The funnel is buried in the soil within the root zone of the crop. While irrigation, water moves downwards through the root zone and the water converges inside the funnel. The soil at the base becomes so wet that water seeps out of it, passes through a filter, and is collected in a reservoir and lifts a float and activates a magnetically latched indicator to pop up [8]. At this time, irrigation will be stopped [7]. Wetting Front Detectors are usually placed in pairs, about one-third and two-thirds down the active root zone.

Early studies have showed that the use of WFD as irrigation scheduling tool decreases the irrigation time, number of irrigation events and increases irrigation interval. Saving of irrigation water at Koga and Meki Irrigation Schemes using WFD for the production of potato and wheat is reported [3]. Scheduling irrigation using WFD also had a positive effect on the yield per plant as well as per unit area of potato, tomato, pepper, and cabbage. Moreover, the use of WFD as irrigation scheduling recorded slightly higher water productivity compared to the farmers practice.

Moreover, the application of blended NPS fertilizer, which substituted DAP in the country influenced the growth and yield of potatoes. Many researchers recorded an increase of plant height, stem number and tuber weight and number and tuber yield of potatoes. Production of potato (*Solanum tuberosum* L.) takes essential place in the highlands of Ethiopia, with an annual production of about 1,141,872 tons harvested from 85,988 ha of land [9].

Potato is a water-stress-sensitive crop. Soil water around the crop roots is especially important in irrigated agriculture as it influences the uptake and movement of nutrients and other agricultural chemicals, which in turn affects crop productivity. Because of the relatively shallow root system, potato requires efficient crop management practices that ensure the availability and uptake of nutrients necessary for improved growth and tuber yield [10]. The purpose of the present study was therefore to evaluate the response of potatoes to WFD as an irrigation scheduling tool and NPS fertilizer.

## 2 Materials and methods

### 2.1 Description of the study area

**Fig. 1:**
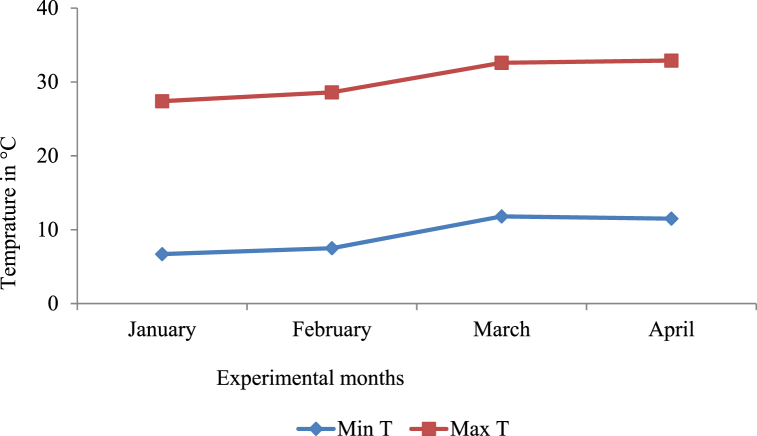
Mean minimum and maximum temperatures during the experimental months.

The study was conducted in the research site of Adet Agricultural Research Center at Koga Irrigation Scheme during the irrigation season from January to April 2016. Koga Irrigation Scheme is one of the modern irrigation schemes developed by the government of Ethiopia to enhance vegetable production in northwest Ethiopia. The command area of the irrigation scheme is about 7000 ha where vegetables including onions, potatoes, tomatoes, pepper, cabbage, carrots, and etc. are produced by smallholder farmers using furrow irrigation system. Cereal crops like wheat, maize, and etc. are also produced during the main rainy season in the scheme [11]. The topography of the irrigation scheme is gentle slope and it is located at 11° 10′ N to 11° 25′ N latitude and 37° 02′ E to 37° 17′ E longitude with the average altitude of about 1960 m above sea level. According to the report of Bahir Dar Meteorology Station (unpublished), the experimental site received the mean rainfall of about 0.08 mm during the experimental period (January to April 2016). The mean maximum and minimum temperatures during the same period were 30.4 and 9.4 °C, respectively (Fig. 1).

To know some of the soil physicochemical properties of the experiment site, soil samples at the depth of 0–30 cm were collected randomly from the entire experimental field of 20 spots, within the experimental field in a zigzag pattern before sowing. A soil composite was made and some properties of the experimental soil were analyzed in Amhara Design and Supervision Works Enterprise soil laboratory.

The collected soil samples were air-dried in wooden trays, ground, and sieved to pass through a 2 mm sieve to exclude components other than soil. The pH of the soil was determined by diluting the soil with water in 1:2.5 ratios. After equilibrating the solution for 2–3 h, the suspension was filtered and the pH was measured by a glass electrode. Organic carbon content of the soil was determined based on the oxidation of organic carbon on acid dichromate medium following the Walkley and Black method as described by Sullivan et al. [12]. Total nitrogen (TN) contents of the samples were determined by Micro-Kjeldahl method [12] and soil cation exchange capacity (CEC) was determined by ammonium acetate method [13]. Salinity of the sample soils was measured as electrical conductivity (EC) and expressed as decisiemens (ds/m) as described by Rhoades et al. [14]. Available phosphorus (Av. P) was also determined using Olsen method as described by Olsen and Dean [15]. Available sulphur in the sample soil was determined using the turbidimetric procedure as described by Sinclair [16]. Particle size (soil texture) was determined by using the hydrometer method of Day [17].

According to the analysis results, the soil of the experimental site was clay loam in its textural classification with high exchangeable Al^3+^ and moderately acidic with a pH value of 5.83. The organic matter content of the experimental soil was 2.35%. The soil has also 5.25 ppm available phosphorous and 0.12% total nitrogen contents (Table 1). Based on the results of analysis, the soil of the study area is generally suitable for the production of potatoes.

**Table 1:** Physico-chemical properties of experimental soil at Koga Irrigation Scheme.

Soil class	pH (H2O) 1:2.5	EC (dS/m)	CEC (cmol(+)/kg)	OM (%)	TN (%)	Av. P (ppm)	Av. S (mg kg^−1^
Clay loam	5.34	0.04	46.00	2.35	0.12	5.25	16.00

EC = Electrical conductivity; CEC = Cation exchange capacity; OM = Organic matter; TN = Total nitrogen; Av. P = Available phosphorous, Av. S = Available sulphur.

### 2.2 Irrigation water application and scheduling

Furrow irrigation was used for the application of water. Irrigation water applied per each irrigation event was measured using a ¾ inch plastic tube where the ditch from head to furrow was lined with geo-membrane to prevent percolation of irrigation water. The amount of water discharged from the plastic tube was determined by calibrating at a uniform velocity and constant head of water supply. For that purpose, water passed through the plastic tubes was collected into a graduated container and the time required to fill the container was recorded using a stopwatch. Dividing the total volume of water to the total time required expressed the discharge of the plastic tube.

The crop water requirement (CWR) and frequency of irrigation water was determined by CROPWAT model using software version 8. The required amount of water was then applied using a stopwatch at ten days intervals. In the case of WFD, two WFDs were buried at 20 cm (yellow-colored indicator) and 40 cm (red-colored indicator) depths of the active root zone of potato, which were placed at 1 m from the end of each plot. In this case, irrigation was stopped when the shallow indicator at 20 cm depth was popped up and the next irrigation was done one day after the yellow indicator stayed down after pressing the indicator down [3,7,8].

### 2.3 Test crop and NPS fertilizer application

Belete variety of potato developed and released by Holetta Agricultural Research Centre was used as a test crop. Healthy seed tubers of the variety which were produced by Adet Agricultural Research Center were used. According to MANR [18], the variety is a high yielder, relatively disease tolerant and adapted to a wide range of altitudes.

Blended NPS fertilizer (19% N, 38% P_2_O_5_ and 7% S) was used in the study. The fertilizer has been recently substituted DAP as a source of phosphorous in Ethiopian crop production system. Based on the treatments, the whole amount of NPS fertilizer was applied at the time of planting.

### 2.4 Experimental treatment and design

The treatments consisted of 6 levels of NPS (0, 90.8, 136.2, 181.6, 227.4 and 272 kg ha^−1^) and two irrigation frequency determination methods (Wetting Front Detector (WFD) and Crop water requirement (CWR). The base for NPS fertilizer levels was the blanket recommendation of DAP (150 kg ha^−1^), which is represented by the treatment 181.6 kg ha^−1^ NPS in the present study. The experiment was laid down in Randomized Complete Blok Design in a factorial arrangement with three replications. The size of the plot was 3 m × 6 m. Each plot contained four rows (75 cm apart) where each row accommodated 20 plants with 30 cm intra-row spacing. An empty space of 100 cm and 50 cm were maintained between blocks and plots, respectively, for ease of agronomic practice. Harvesting was done from the net plot area of 8.1 m^2^ by excluding the two outside rows and one plant from each side of the plot. All management activities except fertilizer and water applications were applied uniformly for each of the experimental plots as recommended by EIAR [19].

### 2.5 Data collection and analysis

#### 2.5.1 Phenological parameters

Days to flowering was determined by counting the number of days elapsed from the date of planting to the date when 50% of the plants in the plot started flowering through visual observation. Days to maturity was also determined by counting the number of days elapsed from the date of planting to the date when more than 90% of the plants in a plot dried and attained physiological maturity.

#### 2.5.2 Growth parameters

Plant heights of ten randomly taken plants grown in the net plot area were measured from the ground level to the tip of the main stem using rulers at physiological maturity and the mean values were computed and used for further analysis. Similarly, the number of main stems sourced from the original tuber of five randomly taken hills/plants was counted 45 days after planting and mean values computed and used for further analysis.

#### 2.5.3 Yield parameters

Mean tuber weight in gram was computed by weighing ten randomly taken tubers that were harvested from the net plot area. Tuber yield was determined by weighing tubers harvested from the net plot area and expressed in a hectare basis.

#### 2.5.4 Determination of irrigation water productivity

Irrigation water productivity is generally defined as crop yield per water used for its production [20]. Irrigation water productivity was therefore calculated as fresh weight (kg) obtained per volume of irrigation water applied (m^−3^) using equation (1) as indicated by Howell et al. [20].

(1) $$\text{Irrigation}\;\text{water}\;\text{use}\;\text{efficiency}=\frac{Yield\left(kg\right)}{water\;applied\;\left(m^{3}\right)}$$

#### 2.5.5 Partial factor productivity

It is a simple production efficiency expression, calculated in units of crop yield per unit of nutrient applied using equation (2) as indicated by Dobermann [21] and Fixen et al. [22].

(2) $$Partial\;factor\;productivity=\frac{Y}{F}$$

Where y is the yield (kg) of the crop with nutrient applied and f is the amount of NPS fertilizer applied (kg).

#### *2.5.6* *Agronomic* efficiency

Agronomic efficiency reflects the direct production impact of an applied fertilizer and relates directly to economic return. It is a unit of yield increase per unit of nutrient applied and calculated using equation (3) as indicated by Dobermann [21] and Fixen et al. [22].

(3) $$Agronomic\;efficiency=\frac{\left(Y-Y0\right)}{F}$$

Where Y is the yield (kg) of the crop with NPS applied; Y0 is the yield (kg) without NPS applied and f is the amount of NPS applied (kg).

#### 2.5.7 Data analysis

The collected data of all parameters of potato were subjected to two-way Statistical Analysis of Variance (ANOVA) using Statistical Analysis Software [23] version 9.2. Whenever treatment effects were significant, mean separations were conducted using Least Significant Difference (LSD) depending on ANOVA results [24].

### 2.6 Economic analysis

To determine the economic feasibility of the treatments, economic analysis in the form of partial budget analysis and marginal rate of return was done according to the procedures developed by CIMMYT [25]. The gross benefit was calculated by multiplying the adjusted marketable tuber yield with the average farm gate price of potato where the marketable yield was downscaled by 10%. Net benefit and marginal rate of returns of the treatments were calculated based on equation (4) and equation (5), respectively. Marginal rate of return is used to compare the relative profitability of treatment options, where the lowest accepted marginal rate of return in developing countries like Ethiopia is 100% [25]. The MRR was calculated after arranging the treatments in ascending order of the variable costs. Treatments that have net benefits lower or equals to the previous treatment are dominated and were excluded from the MRR analysis as indicated by CIMMYT [25].

The costs of NPS and the labor requirement for placement of fertilizer and application of water were considered as the variable costs of the experiment. Moreover, the market prices of fertilizer and tubers and cost of labor were taken from the market assessment during the experimental period.

(4) NB=GB−VC

(5) $$\text{MRR}\;\left(\%\right)=\left(\frac{\Delta \text{NB}}{\Delta \text{VC}}\right)*100$$

Where:

NB = Net benefit; GB = Gross benefit; VC = variable cost; MRR = Marginal rate of return; ΔNB = change in net benefit and ΔVC = change in variable cost.

## 3 Results and discussion

### 3.1 Plant height

Plant height of potato was influenced by the rates of NPS fertilizer and irrigation scheduling methods as well as their interaction (P < 0.01). Scheduling irrigation using wetting front detector significantly increased plant height while NPS fertilizer up to 227.4 kg ha^−1^ increased the height of potato plants. Further increase of NPS fertilizer rate however had not improved the plant height. Plants supplied with 227.4 kg ha ^−1^ NPS and irrigated using wetting front detector as well as those supplied with 272 kg ha ^−1^ NPS and irrigated using crop water requirement recorded the longest potato plants with the values of 65.2 cm and 64.8, respectively, which were statistically similar (Table 2). On the other hand, potato plants grown without NPS fertilizer and irrigated based on crop water requirement were shorter (40.3 cm) than those plants grown without NPS fertilizer but irrigated based on wetting front detector method (42.7 cm).

**Table 2:** Effects of irrigation scheduling methods and NPS fertilizer rate on the growth and phenology of potato.

Treatment	PH (cm)	SN (count/hill)	DOF (days)	DOM (days)
Irrigation scheduling methods				
Wetting front detector	55.2^a^	7.9^a^	76.3^a^	108.1^a^
Crop water requirement	51.3^b^	7.4^b^	74.5^b^	107.3^b^
LSD (P = 0.05)	0.28	0.14	0.58	0.37
NPS rates (kg ha^−1^)				
0.0	41.5^e^	5.5^f^	67.2^e^	103.0^e^
90.8	47.6^d^	6.6^e^	71.2^d^	106.0^d^
136.2	52.1^c^	7.5^d^	75.2^c^	107.3^c^
181.6	53.9^b^	7.9^c^	77.7^b^	108.8^b^
227.4	62.0^a^	8.7^b^	78.3^b^	109.3^b^
272.0	62.5^a^	9.8^a^	82.8^a^	111.5^a^
LSD (P = 0.05)	0.49	0.24	1.0	0.63
Irrigation scheduling methods x NPS rates				
Wetting front detector				
0.0	42.7^i^	5.8^h^	67.7^g^	104.0^g^
90.8	50.1^g^	6.6^g^	72.7^e^	105.3^f^
136.2	53.7^e^	7.7^e^	76.3^d^	107.7^de^
181.6	55.0^d^	8.1^cd^	78.0^c^	109.7^b^
227.4	65.2^a^	9.0^b^	78.7^c^	109.7^b^
272	64.8^a^	10.4^a^	84.3^a^	112.0^a^
Crop water requirement				
0.0	40.3^j^	5.2^i^	66.7^g^	102.0^h^
90.8	45.0^h^	6.5^g^	69.7^f^	106.7^e^
136.2	50.4^g^	7.2^f^	74.0^e^	107.0^de^
181.6	52.7^f^	7.7^de^	77.3^cd^	108.0^dcd^
227.4	58.9^c^	8.4^c^	78.0^c^	109.0^bc^
272	60.2^b^	9.3^b^	81.3^b^	111.0^a^
LSD (P = 0.05)	0.77	0.35	1.38	1.05
CV (%)	0.77	2.64	1.11	0.49
SE±	0.41	0.20	0.83	0.53

PH = Plant height; SN = Stem number; DOF = Days to flowering; DOM = Days to maturity; LSD = Least Significant Deference; CV = Coefficient of variance; SE = Standard error; Means within the column followed by the same letter/s are not significantly different at p = 0.05.

The use of wetting front detector method for irrigation scheduling increases the growth and development of different plants by improving the nutrient use efficiency [3], which is also indicated in the present study. Potato plants irrigated based on wetting front detector method required the supply of 227.4 kg ha^−1^ NPS fertilizer and while those irrigated based on crop water requirement method required 272 kg ha^−1^ NPS fertilizer to attain the same plant heights. Enhancement of growth and development of different vegetables using NPS fertilizer and wetting front detector tools is also reported by different researchers [[26], [27], [28], [29]].

### 3.2 Stem number

NPS fertilizer rates and irrigation scheduling methods as well as their interaction influenced the number of stems per hill of potato (P < 0.01). Wetting front detector method of irrigation scheduling recorded more stem count per hill. Similarly, increasing NPS fertilizer rates increased the stem number of potatoes per hill. Plants supplied with 272 kg ha^−1^ NPS and irrigated based on wetting front detector produced the highest number of stem count per hill (10.4). On the other hand, the same rate of NPS fertilizer combined with crop water requirement method recorded the second highest number of stem count per hill (Table 2).

The results of the present study clearly showed the increase of stem count per hill through the application of NPS fertilizer, which could be associated with the growth promoting effects of plant nutrients supplied with the blended fertilizer [29,30]. The increase in potato stem number at higher rates of NPS and irrigation using wetting front detector was more pronounced compared to the crop water requirement method. This is due to the fact that wetting front detector method detects the moisture at the root zone of the plants and thus it reduces the leaching of nutrients and enhances the nutrient use efficiency of the plants.

### 3.3 Days to flowering and maturity

Blended NPS fertilizer and irrigation scheduling methods (P < 0.01) as well as their interaction (P < 0.05) influenced days to flowering of potato. Similarly, days to maturity was influenced by the main effects of the two factors and their interaction (P < 0.01). Plants irrigated through the wetting front detector method flowered and matured relatively late compared to those irrigated using crop water requirement method (Table 2). Delayed flowering and maturity was also observed with increased NPS fertilizer, which is clearly associated with the enhanced vegetative growth that in turn prolongs the flowering and maturity of plants. Potato plants grown without the supplement of NPS fertilizer flowered and matured early. Potato plants supplied with 272 kg ha^−1^ NPS and irrigated with wetting front detector method flowered and matured late at 84.3 and 112 days after planting, respectively, while those supplied with the same rate of NPS and but irrigated with crop water requirement scheduling method flowered and matured a bit earlier.

Application of blended NPS fertilizer and irrigation using wetting front detector as an irrigation frequency determination method enhanced the growth and development of potato plants as indicated in terms of plant height and stem number, which in turn clearly prolongs the date of flowering and maturity of potato. Prolonged flowering and maturity of potato was also reported by different researchers [28,29,31]. Similarly, enhanced growth and productivity of potato using wetting front detector method was reported by Schmitter et al. [3].

### 3.4 Tuber weight and yield of potato

**Fig. 2:**
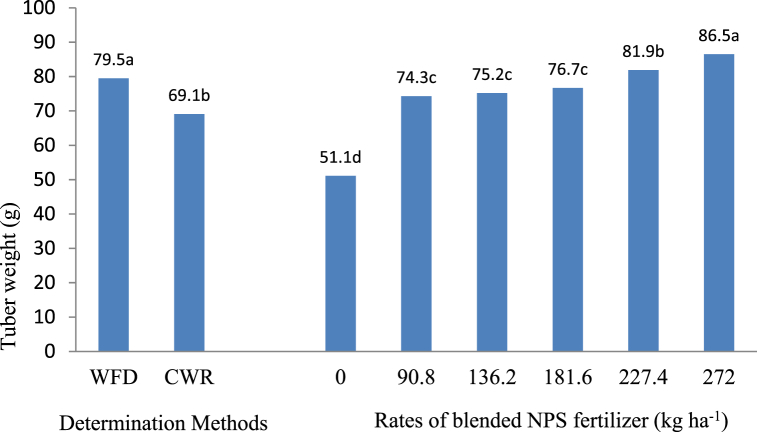
Potato tuber weight as influenced by irrigation frequency determination method and rates of blended NPS fertilizer. Note: WFD = Wetting front detector; CWR = Crop water requirement; Means within the group followed by the same letter/s are not significantly different at p = 0.05.

**Fig. 3:**
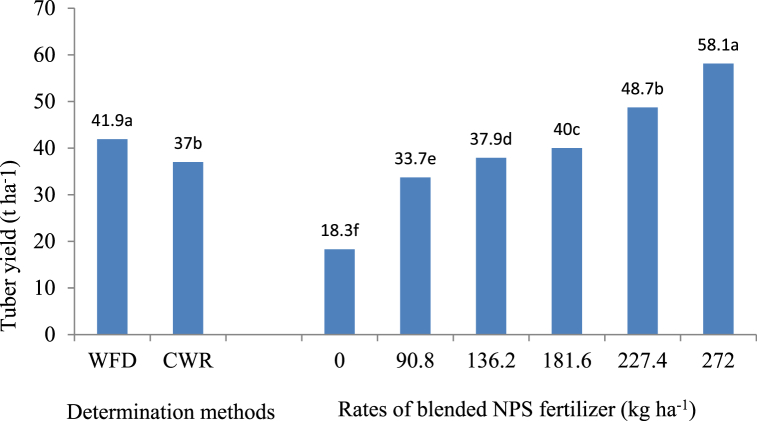
Effect of irrigation frequency determination method and rate of blended NPS fertilizer on tuber yield of potatoes Note: WFD = Wetting front detector; CWR = Crop water requirement; Means within the group followed by the same letter/s are not significantly different at p = 0.05.

Tuber fresh weight was influenced by NPS fertilizer rate and irrigation frequency determination method (P < 0.01), while their interactions did not influence this parameter (P > 0.05). Potato plants irrigated with wetting front detector scheduling method recorded the biggest tubers (79.5g) while those plants irrigated using crop water requirement scheduling produced the smallest tubers (69.1 g). NPS at the rate of 272 kg ha^−1^ recorded the biggest potato tubers followed by 227.4 kg ha^−1^ NPS. On the other hand, plants without NPS fertilizer produced the smallest tubers (Fig. 2). Similarly, tuber yield was influenced (p < 0.01) by the rate of NPS fertilizer and frequency determination method. However, the interaction of these factors did not influence the tuber yield (p > 0.05). The highest tuber yield was obtained when irrigation water was applied based on wetting front detector method and by the application of 272 kg ha^−1^ NPS fertilizer as indicated in Fig. 3.

Tuber weight and yield of potatoes are among others influenced by soil fertility and the type and quantity of the applied fertilizer. In this regard, the application of NPS fertilizer in the present study improved the weight and tuber yield of potato, which is clearly associated with the combined effects of plant nutrients that are found in the applied fertilizer. Nitrogen, phosphorus, and potassium are essential nutrients for the growth and productivity of a given crop including potatoes. Nitrogen is an essential nutrient for vegetative growth and photosynthetic activity that converts solar energy to carbohydrates and stored in the tubers. Phosphorus enhances early root and shoot development, provides energy for plant processes such as ion uptake and transport. Potassium also helps regulate the opening and closing of the stomata, which regulates the exchange of water vapor, oxygen, and carbon dioxide. Similar findings were also reported by different researchers where application of blended NPS fertilizer improved the fresh weight and tuber yield of potatoes [[28], [29], [30], [31], [32]].

The fact that wetting front detector measures the moisture at the root zone of plants [7], the technology helps to reduce leaching of plant nutrients beyond plant root zones and thus reduces the amount of fertilizer needed for the production of crops. Moreover, it also improves the nutrient use efficiency by enhancing the crop yield [3] as observed in fresh weight and tuber yield of potato in the present study.

### 3.5 Irrigation water productivity

Data on irrigation water productivity are presented in Table 3. Crop water requirement scheduling method required higher amounts of water (4764 m^−3^) than wetting front detector method (4580 m^−3^) (data not indicated). Wetting front detector scheduling method recorded significantly highest irrigation water productivity compared to crop water requirement scheduling method (P < 0.01). However, the irrigation water productivity did not differ (P > 0.05) for irrigation scheduling method and NPS fertilizer interactions. Irrigation water productivity was significantly increased with increased NPS fertilizer where the highest efficiency was recorded at 272 kg ha^−1^ NPS. Treatment without NPS fertilizer recorded the lowest irrigation water use efficiency.

**Table 3:** Irrigation water productivity, partial factor productivity and agronomic efficiency of potato as influenced by irrigation scheduling methods and NPS fertilizer rates.

Treatment	Partial factor productivity	Agronomic efficiency	Irrigation water productivity (kg of potato m^−3^ water)
Irrigation scheduling methods			
Wetting front detector	275.2^a^	143.8	9.1^a^
Crop water requirement	243.6^b^	141.2	7.8^b^
LSD (P = 0.05)	6.1	6.6	0.16
NPS rates(kg ha^−1^)			
90.8	371.4^a^	169.5^a^	3.9^f^
136.2	278.0^b^	143.4^bc^	7.2^e^
181.6	220.0^c^	119.1^d^	8.1^d^
227.4	214.3^c^	133.6^c^	8.6^c^
272.0	213.4^c^	146.8^b^	10.5^b^
LSD (P = 0.05)	9.60	10.40	12.40^a^
CV (%)	3.05	6.01	0.28
SE±	7.90	8.56	2.79

LSD = Least Significant deference; CV = Coefficient of variance; SE = Standard error; Means within the column followed by the same letter/s are not significantly different at p = 0.05.

Wetting front detector scheduling method is helpful for the effective use of irrigation water and reduction of its wastage during application and thereby increases irrigation water use efficiency. In this regard, Ati et al. [33] reported that the use of effective water management practices will help to address the global water crisis in the future thereby reducing wastage. Results of different researchers are also confirmed the results of the present study [4,7,34]. Nutrients contained in NPS fertilizer (nitrogen, phosphorous, and potassium) are essential nutrients taken up by potato plants in the greatest quantity and improve the growth and yield of potato and thereby improve the irrigation water use efficiency.

### 3.6 Partial factor productivity and agronomic efficiency

Partial factor productivity was influenced by irrigation scheduling method, NPS fertilizer rate as well as their interaction (P < 0.01). Wetting front detector method recorded the highest partial factor productivity compared to crop water requirement method. Increasing the NPS rates reduced partial factor productivity (Table 3). Highest partial factor productivity was recorded when potato plants were supplied with 90.8 kg ha^−1^ and irrigated using wetting front detector method (Table 4). Increasing the NPS rate reduced the partial factor productivity in both irrigation scheduling methods where wetting front detector method combined with NPS rates generally recorded the highest partial factor productivity compared to the respective combination of crop water requirement with NPS fertilizer rates.

**Table 4:** Interaction effect of NPS fertilizer rate and irrigation frequency determination method on partial factor productivity of potato.

NPS rate (kg ha^−1^)	Partial factor productivity (kg potato/kg NPS)	
	Wetting front Detector	Crop water requirement
90.8	398.8^a^	338.1^b^
136.2	297.9^c^	258.0^d^
181.6	230.7^e^	209.4f^g^
227.4	228.2^e^	200.3^g^
272.0	220.4^ef^	206.4^fg^
LSD (P = 0.05)	14.7	
CV (%)	3.05	
SE±	7.90	

LSD = Least Significant deference; CV = Coefficient of variance; SE = Standard error; Means within the column followed by the same letter/s are not significantly different at p = 0.05; Means followed by the same letter/s are not significantly different at p = 0.05.

Agronomic efficiency was not significantly influenced (p > 0.05) by the irrigation scheduling methods and their interaction with NPS rates. The NPS rates on the other hand influenced the agronomic efficiency where the lowest rates recorded the highest agronomic efficiency (Table 3).

Nutrient use efficiency is a critical concept for evaluating crop production systems and can be greatly impacted by fertilizer management as well as soil- and plant-water relationships (22 Fixen et al., 2015). The NPS efficiency conducted in terms of partial factor productivity was higher in wetting front detector method than crop water requirement. According to 22 Fixen et al. (2015), higher values of partial factor productivity indicate the responsiveness of the soil and plant to added nutrients and suggest that nutrient supply is likely limiting productivity. On the other hand lower levels of partial factor productivity are indication for less responsive soils or over application of nutrients [22]. In wetting front detector method, irrigation will be stopped as the irrigation water reached the root zones of plants [3,7]. Such conditions increase nutrient availability and reduce the leaching of the nutrients beyond the root zone, which in turn increase performance of the applied fertilizer as indicated in the present study.

Agronomic efficiency as one form of nutrient use efficiency closely reflects the direct production impact of an applied fertilizer and relates directly to economic return. Lower values of agronomic efficiency indicated by higher NPS rates in the present study suggest changes in management for the improvement of crop response or reduction of inputs, while high values indicate the affectivity of the applied fertilizer [22].

### 3.7 Partial budget analysis

Partial budget analysis is a method of organizing experimental data and information about the costs and benefits of various alternative treatments. The cost benefit analysis in the present study was conducted based on the procedures described by CIMMYT [25]. Gross benefit of each treatment was calculated by multiplying the downscaled (10%) tuber yield with field prices (5.0^−1^) of potato during the experimental period. Price of NPS fertilizer (15.5 ETB kg^−1^) and labor costs for application of fertilizer and water application (100 ETB per man-day) were considered as variable costs for calculating the net benefit of each treatment.

Accordingly, the highest net benefit was obtained by the treatment combination of 272 kg ha^−1^ NPS and wetting front detector irrigation scheduling method with the value of 236,591.7 ETB ha^−1^ while the lowest net benefit was recorded at 0 kg ha^−1^ NPS fertilizer and irrigation based on crop water requirement method (Table 5). All none-dominated treatment combinations recorded acceptable marginal rates (>100%). However, the treatment combination of 272 kg ha^−1^ NPS and wetting front detector method recorded the highest net benefit which is more profitable compared to the other treatment combinations (Table 6).

**Table 5:** Partial budget analysis of potato as influenced by irrigation frequency determination method and NPS fertilizer rate.

Treatment combinations	Adjusted tuber yield (t ha^−1^)	Gross field benefit (ETB ha^−1^)	Total variable costs (ETB ha^−1^)	Net benefit (ETB ha^−1^)	Rank
0 NPS x WFD	18.5	92,610.0	19,753.1	72,856.9	11
0 NPS x CWR	14.5	72,450.0	14,812.8	57,637.2	12
90.8 NPS x WFD	32.6	162,945.0	26,716.0	136,229.0	8
90.8 NPS x CWR	28.1	140,580.0	21,775.8	118,804.2	10
136.2 NPS x WFD	36.5	182,610.0	28,160.5	154,449.5	6
136.2 NPS x CWR	31.6	158,130.0	23,220.2	134,909.8	9
181.6 NPS x WFD	37.7	188,505.0	29,975.3	158,529.7	5
181.6 NPS x CWR	34.2	171,135.0	25,035.0	146,100.0	7
227.4 NPS x WFD	46.7	233,505.0	31,425.9	202,079.1	3
227.4 NPS x CWR	41.0	205,020.0	26,485.7	178,534.3	4
272 NPS x WFD	54.0	269,820.0	33,228.3	236,591.7	1
272 NPS x CWR	50.5	252,585.0	28,288.1	224,296.9	2

WFD = Wetting front detector; CWR = Crop water requirement; ETB = Ethiopian Birr.

**Table 6:** Analysis of marginal rate of return.

Treatment combination	Variable cost (ETB ha^−1^)	Net benefit (ETB ha^−1^)	Dominance	MRR (%)
0 NPS x CWR	14,812.8	57,637.2		
0 NPS x WFD	19,753.1	72,856.9		308.1
90.8 NPS x CWR	21,775.8	118,804.2		2271.6
136.2 NPS x CWR	23,220.2	134,909.8		1115.0
181.6 NPS x CWR	25,035.0	146,100.0		616.6
227.4 NPS x CWR	26,485.7	178,534.3		2235.9
90.8 NPS x WFD	26,716.0	136,229.0	Dominated	
136.2 NPS x WFD	28,160.5	154,449.5	Dominated	
272 NPS x CWR	28,288.1	224,296.9		2539.0
181.6 NPS x WFD	29,975.3	158,529.7	Dominated	
227.4 NPS x WFD	31,425.9	202,079.1	Dominated	
272 NPS x WFD	33,228.3	236,591.7		248.9

WFD = Wetting front detector; CWR = Crop water requirement; ETB = Ethiopian Birr.

## 4 Conclusion and recommendation

The results of the present study showed that NPS fertilizer rate and irrigation frequency determination methods influenced growth, yield, irrigation water productivity, and fertilizer use efficiency of potatoes. Wetting front detector method combined with 272 kg ha^−1^ NPS fertilizer recorded the highest plant height and stem number of potatoes. Wetting front detector improved tuber weight, tuber yield and irrigation water productivity compared to crop water requirement method. Highest rate of NPS fertilizer also produced the highest tuber weight, tuber yield and irrigation water productivity. Similarly, the combination of wetting front detector method with all rates of NPS fertilizer recorded enhanced partial factor productivity compared to the treatment combinations of crop water requirement with the respective rates of NPS fertilizer. Wetting front detector combined with 272 kg ha^−1^ NPS recorded the highest net benefit with an acceptable marginal rate of return. The use of wetting front detector as an irrigation water frequency determination method and application of NPS fertilizer at the rate of 272 kg ha^−1^ helps to enhance growth, tuber yield, irrigation water productivity, and fertilizer use efficiency of potato at Koga Irrigation Scheme and areas with similar agro-ecologies.

## Author contribution statement

Melkamu Alemayehu: Conceived and designed the experiments; Analyzed and interpreted the data; Wrote the paper.

Minwyelet Jemberie: Performed the experiments; Analyzed and interpreted the data.

Yigizaw Dessalegn: Analyzed and interpreted the data; Contributed reagents, materials, analysis tools or data.

### Additional information

No additional information is available for this paper.

## Data availability statement

Data will be made available on request.

## Declaration of competing interest

The authors declare that they have no known competing financial interests or personal relationships that could have appeared to influence the work reported in this paper.
